# A Hospital Performance Assessment Model Using the IPOCC Approach

**DOI:** 10.4314/ejhs.v31i3.10

**Published:** 2021-05

**Authors:** Roya Malekzadeh, Ghasem Abedi, Edris Hasanpoor, Matina Ghasemi

**Affiliations:** 1 Department of Public Health, Mazandaran University of Medical Sciences, Sari, Iran; 2 Department of Public Health, Health Sciences Research Center, Mazandaran University of Medical Sciences, Sari, Iran; 3 Department of Healthcare Management, Research Center for Evidence-Based Health Management, Maragheh University of Medical Sciences, Maragheh, Iran; 4 Faculty of Business and Economics Department, Girne American University, Kyrenia

**Keywords:** performance assessment, health management, organizational excellence model

## Abstract

**Background:**

Developing a practical model to assess hospital performance improves the quality of services and leads to patient satisfaction. This study aims to develop and present such a model using the IPOCC (Input, Process, Output, Control and Context) approach.

**Methods:**

This study used a mixed-method research. The statistical population of the qualitative part included 27 experts who were purposefully selected and the sampling process was continued by the snowball method until the data saturation was reached. The quantitative part included 334 managers at different levels within a hospital, who were selected by a random sampling method based on Cochran's formula.

**Results:**

The hospital evaluation model has 5 dimensions with 20 factors: input (human, financial, physical, information and equipment), process (treatment, para-clinical, prevention, management, and leadership processes), outcome (patient, staff and community outcomes and key performance index), control (internal control, external control), context (hospital culture, hospital status, the role of evaluators and community conditions). The value of chi-square was 4689.154, the degree of freedom was 2385, and the ratio of chi-square to the degree of freedom in the model was 1.966, which is an acceptable value. The values obtained from CFI, GFI, and IFI fit indices were acceptable. The SRMR index was 0.1130.

**Conclusions:**

Using a performance assessment model along with the IPOCC approach evaluates hospital processes and the output obtained from the proper implementation of these processes in all areas. The areas include the hospital provided services like the control and context, or the traditional perspectives like physical, human, financial, and equipment resources.

## Introduction

Performance measurement is a numerical scale that measures the quality of activities performed in an organization (1). Global changes have created new challenges in the field of health (2).

Hospital complexity (3), the increasing costs of health care, and the vitality of the provided care are among the factors that encouraged health care organizations to apply changes in performance assessment (4). Developing an appropriate and practical model for evaluating hospital performance can potentially result in accountability, service quality improvement, and patient satisfaction (5). Where hospital performance was not assessed, no attempts had been made to improve the performance (6). Therefore, assessment and improvement of performance are two sides of the same coin, and making an effort to accomplish the first goal gives rise to changes in the second one (7).

There have been serious difficulties in developing a hospital performance assessment system (8). The necessity for a performance assessment system at any organization is such that the lack of an assessment system in different dimensions of any organization is considered as one of the symptoms of the organization's disease (9). Currently, performance assessment in Iranian hospitals is mainly focused on legal requirements and accreditation standards. Other evaluations are performed sporadically in different departments of hospitals, on a case-bycase basis (10), which in turn give rise to the adoption of various hospital performance assessment models. Many of these models are unsatisfactory because of their narrow focus and one-dimensional results of hospital performance (11). Thus, the lack of combined markers may be misleading, and to improve these complex processes, a combination of different input, process, and outcome measures will be required (6).

Some new and different methods for evaluating the performance of the hospital include the organizational excellence model, the balanced scorecard (BSC), and accreditation (12). Taking into consideration the indicators leading to a comprehensive improvement of the performance of the organization is of great importance (12, 13).

Nasiripour et al.'s study on Iranian hospitals' performance revealed that there is no balanced perspective on performance assessment (14). Taslimi and Zayandeh also mentioned the lack of evaluation of different aspects of hospital performance and the absence of stakeholders as the challenges of designing a hospital performance assessment system (6). Research by Sanayeei et al. indicated that the performance of the studied hospital was far from optimal (10).

According to the IPOCC model, each system will produce an expected output, given a certain input implemented by a series of processes on the inputs and control measures. Some actions in this model are in the context of all actions and steps and affect all parts of the system. In Canada, Nippak et al. evaluated hospital performance with selected criteria using a SIPOC model. They considered the use of this model to help develop comprehensive performance indicators(15). Malekzadeh et al. evaluated the hospital's performance using “EFQM” and “IPOCC” models. The results showed that the IPOCC model helps provide a comprehensive classification for all aspects of hospital performance such as input, process, outcome, control, and service delivery, which explores the strengths and weaknesses of the tool. Assess existing performance helps and allows the evaluation of hospital performance in a principled manner(16). It is extremely evident that hospital performance assessment is very useful and shows how the activities have been performed and the resources have been used and also provides the information required by managers to evaluate and monitor the current activities of the hospital (17).

So far, studies conducted on hospital performance assessment have been based on a quality model or some of its subsequent dimensions, not a comprehensive model to achieve the relevant goal. The current study entitled “Develop and present a hospital performance assessment model using the IPOCC approach” aimed to present a comprehensive performance assessment model that considers input, process, output, control, and context dimensions altogether, and provides a context for assessing performance within a principled, systematic framework in the healthcare sector.

## Methods

Designed as mixed-method research, the present study was composed of both qualitative and quantitative methods based on five phases including “comprehensive text review, the qualitative phase of expert interviewing, expert panel formation, quantitative phase, and model development”. The statistical population of the qualitative section included professors of health services management, hospital managers, national accreditation assessors, and quality improvement officers. The statistical population of the quantitative section included top, middle, and operational managers of the hospitals in Mazandaran province who met the inclusion criteria.

In the qualitative section, samples were purposefully selected. First, the experts were identified, and then, the snowball sampling method was applied. Sampling (interviews as samples) was continued until the data saturation was reached, with 27 interviews.

The data analysis also followed qualitative content analysis methods. To analyze the data and to measure the reliability of the results, the interviews were recorded by the research team (the researcher and two colleagues) and later were examined and analyzed, separately. Similarities in their results showed the reliability of the results obtained. To measure the validity of the method, not only were the comprehensive, organized, and basic themes selected based on theoretical foundations and literature reviews before approval but also were the opinions and views of a group of experts applied.

To ensure the trustworthiness and rigor of the study, Guba and Lincoln's proposed criteria were used (18). The researchers sought to improve the credibility of the research by prolonged engagement, sufficient interaction with participants, collection of valid information, and member check. To increase the dependability of the data, measures such as step-by-step replication and data collection and analysis were taken. Moreover, data were reviewed by the supervisors, consultants, and experts. To increase the confirmability of the data, the faculty members confirmed the data, and their additional comments were considered. Data transferability was achieved by attempting to provide a detailed description of the research report to provide a basis for assessing the applicability of the results in other settings. Moreover, the quotations of the participants were kept as authentic to the original words as possible.

In the quantitative part, we used simple random sampling to select managers in different levels of hospitals and had the inclusion criteria studied. Cochran`s formula with an estimation error of 0.05 (d=0.05) and the first type error of 0.001 (α =0.001) determined the sample size be 334.

The inclusion criteria were top, middle, and operational managers with at least 10 years of experience in management and 3 years of experience in performance assessment and activities such as developing strategic and operational plans. The exclusion criterion was the unwillingness of the statistical population to participate in the study.

An attempt was made to prepare a questionnaire based on 75 components obtained from the qualitative content analysis of interviews. For the quantitative measurement of content validity, (both) content validity ratio (CVR) and the content validity index (CVI) were calculated. The content validity of the questionnaire was confirmed based on expert opinion (CVR was more than 0.73 and CVI was more than 0.87 for all items).

A group of 20 experts had participation to explore their opinions regarding the main purpose of the study. The construct validity of the questionnaire was investigated using exploratory factor analysis (EFA) and confirmatory factor analysis (CFA) and 71 items were confirmed.The reliability of all its dimensions was confirmed by Cronbach's alpha coefficient (above 0.70) and compound reliability (above 0.60). The questionnaire was graded on a 5-point Likert scale ranging from not taking adequate efforts (completely agree) to full progress in any area (completely disagree).

This questionnaire covers 20 factors: human resources (3 questions), financial resources (3 questions), physical resources (4 questions), information resources (3 questions), equipment resources (3 questions), treatment processes (6 questions), Para clinical processes (5 questions), prevention processes (4 questions), management and leadership processes (5 questions), patient outcomes (3 questions), staff outcomes (3 questions), community outcomes (4 questions), key hospital outcomes (3 questions), internal control (4 questions), external control (2 questions), hospital culture (3 questions), hospital status (4 questions), the role of evaluators (3 questions) and community conditions (4 questions).

SPSS18 software was used to perform exploratory factor analysis and descriptive statistics on the data, and Amos software was used for confirmatory factor analysis and model fit. The chi-square index, normalized fit index (19), confirmatory fit index (CFI), the goodness of fit index (GFI), Standardized Root Mean Square Residual (RMSEA), adjusted goodness of fit index (AGFI), incremental fit index (IFI), and non-normalized fit index (NNFI) were used to evaluate the adequacy of the model.

**Ethical considerations**: This study is the result of a doctoral dissertation approved by the Ethics Committee of Islamic Azad University, Sari Branch, (ethicscode: IR.IAU.CHALUS.REC.1397.024).

## Results

In the qualitative section, the opinions of 27 participants, who were knowledgeable about the hospital performance assessment, were evaluated. They included 5 professors of health services management, 3 staff managers of the Ministry of Health, 5 hospital managers, 10 national accreditation assessors, and 5 hospital quality improvement officers. Using framework analysis, 5 main themes including input, process, output, control, and context, 20 sub-themes, and 75 sub-contents were extracted for performance assessment.

Then, using exploratory factor analysis (EFA), the main dimensions of the construct designed to measure the desirable variables were examined and discovered. To determine the adequacy of the sample size for factor analysis, the Kaiser-Meyer-Olkin (KMO) Measure of Sampling Adequacy and Bartlett's Test of Sphericity were used, and values greater than 0.7 indicated the appropriate sample size.

As shown in Table 1, the KMO value was 0.886 and the significance level of Bartlett's Test of Sphericity was zero. Therefore, in addition to the adequacy of sampling, the implementation of factor analysis based on the studied correlation matrix could also be justified. Based on the extracted factors and the percentage of explained variance in the hospital performance assessment model, the eigenvalues of the 20 studied factors were greater than one, which together accounted for about 70.83% of the total changes. Based on the results obtained from the varimax rotation, 20 components were extracted. In the questions related to each dimension, the share ratio of questions was greater than 0.50, which indicates these questions were well matched with other questions. The final stage of exploratory factor analysis was naming the factors. In this study, it was done according to the opinions of technical experts as follows: human, financial, physical, information, and equipment resources; therapy, Paraclinical, prevention, and leader processes; patient, employee, community, and hospital performance results; internal control and external control; hospital culture, hospital position in the grading system, the role of evaluators, and the conditions of society.

**Table 1:** Results of the KMO index and Bartlett's test for the hospital performance assessment construct

Construct	Kaiser-Meyer-Olkin (KMO) Measure of Sampling	
	Adequacy and Bartlett's Test of Sphericity	
	KMO	0.886
Hospital performance assessment	Bartlett	14408.517
	df	2775
	P-Value	0.000

To determine the effects of each of the variables and their significance coefficients, second-order factor analysis, standard coefficients, and t-values were used. Figure 1 shows the relationship between the components of the hospital performance assessment model in a standard coefficient mode. As seen in Figure 1 and Table 2, the standard coefficients of input, process, output, control, and context variables were greater than 0.5, the t-values of these variables were more than 1.96, and the P-values of the variables were less than 0.05. The experts concluded that these dimensions were effective in explaining hospital performance assessment.

**Figure 1:**
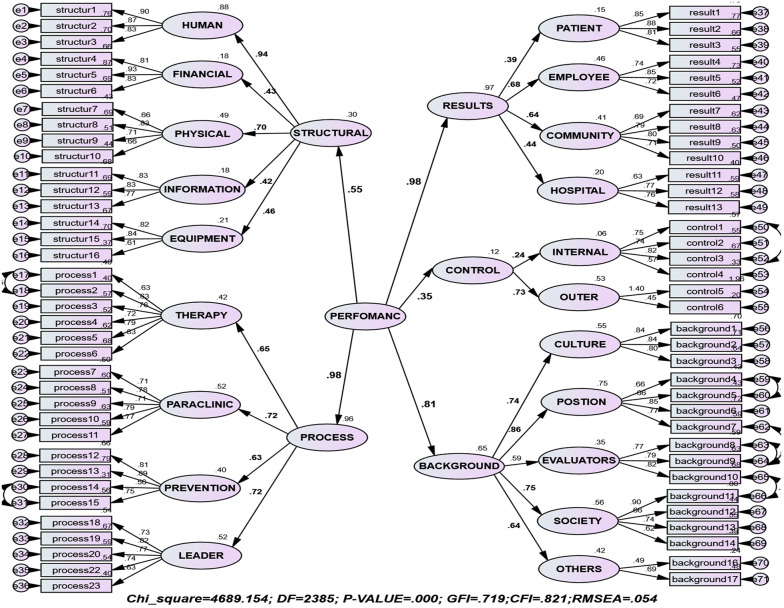
The proposed model in a standard coefficient mode

**Table 2:** Factor analysis (review of the main model) of hospital performance assessment

The explanatory study of factors associated with hospital performance assessment construct	Standard coefficients	t-value	P-Value	Results
Input	0.55	8.711	0.0009	Significant
Process	0.98	6.687	0.0009	Significant
Outcome	0.98	6.176	0.0009	Significant
Control	0.35	6.606	0.0009	Significant
Context	0.81	10.287	0.0009	Significant

Table 3 shows the appropriateness of the final performance assessment model according to the IPOCC approach. The value of chi-square was 4689.154, the degree of freedom was 2385, and the ratio of chi-square to the degree of freedom was 1.966 as an acceptable value. Moreover, the fit indices of the main model, such as CFI (0.82) and GFI (0.81) were all acceptable and appropriate, and the SRMR index was 0.1130. According to the IPOCC approach, input, process, outcome, control and contextual variables were significant as the main dimensions in explaining the hospital performance assessment. According to this model, each system starts with certain *inputs* including human resources, financial resources, physical resources, information resources, and equipment resources. By implementing a series of treatment, Paraclinical, prevention, and leadership *processes* on the inputs and *control* measures (internal control and external control), the expected *output* of the system is achieved in the dimensions of the patient, employee, community, and hospital. Some actions such as hospital culture, hospital position, the role of the assessor, and conditions of society in this model are in the *context* of all actions and steps and affect all parts of the system.

**Table 3:** Fit indices of the proposed research model

Indices	Acceptable value	The value of the research findings	Desirability
Chi-square (*χ*^2^)	-	4689.154	Model approval
P-Value	-	0.000	Model approval
df (degree of freedom)	*df* ≥ 0	2385	Model approval
*χ*^2^/*df*	*χ*^2^/*df* < 3	1.966	Model approval
RMSEA	RMSEA < 0.1	0.054	Model approval
NFI	NFI > 0.8	0.694	Model disapproval
AGFI	AGFI> 0.8	0.799	Model disapproval
GFI	GFI> 0.8	0.819	Model approval
CFI	CFI > 0.8	0.821	Model approval
IFI	IFI > 0.8	0.822	Model approval
SRMR	The closer it is to zero	0.1130	Model approval

## Discussion

The findings of the present study illustrated that the dimensions of hospital performance assessment included structural, process, results, control, and background dimensions. The hospital evaluation model has 5 dimensions and 20 factors including input (human, financial, physical, information and equipment), process (treatment, Paraclinical, prevention, management, and leadership processes), outcome (patient, staff and community outcomes and key performance index), control (internal control, external control), context (hospital culture, hospital status, the role of evaluators and community conditions). Most of the performance assessment models, especially in the hospital setting, have traditionally focused mainly on the adequacy of facilities and employees as well as the appropriateness of the processes (20). Most of these models have focused on Input elements, some on processes measurement, and others on results, and also achieving a balanced measurement of input, process, and output by using hospital performance assessment systems has rarely occurred (21). In the present model, in addition to balanced attention to the dimensions of input, process, result, control and contextual components have also been considered. Because the decision about the performance of the hospital might be correct by considering all the effective factors in providing service.

Donabedian proposed using the triad of structure, process, and outcome indicators to evaluate performance (22). The dimensions of Donabedian's model are consistent with the three dimensions of the present model, and the only difference is the lack of attention to the control and context dimensions. Using the results of internal monitoring by quality control teams from different units and departments of the hospital is effective in identifying strengths and weaknesses and improve the performance of the hospital. Utilizing the feedback of outside observers, such as accreditation inspectors, also helps to plan for the hospital's weaknesses.

Kaplan and Norton (1996) provided the indicators of performance assessment with a balanced scorecard (BSC) model in four perspectives: financial, customer, internal process, and learning and growth (23). Apart from the internal process dimension, other dimensions of the BSC model differ from the dimensions of the present model and are considered in some categories and subcategories. Perhaps one of the reasons for this difference is the origin of the BSC model in the industry. Therefore, modification of processes and customer relationships to improve the financial performance and growth and learning of the organization is considered. The design of the current model is based on the performance of the hospital.

The organizational excellence model evaluates performance based on nine main criteria: “leadership, policy and strategy, employees, partnerships and resources, processes, customer results, employee results, society results, and key performance results” (24). The process dimension is considered as the main dimension of the present model and the dimensions of customer results, employee results, society results, and key performance results in the organizational excellence model are regarded as the results dimension in the present model. Other dimensions are considered as categories and subcategories. Mosadeghrad (2018) proposed National Accreditation of Iran is a systematic model for hospital performance evaluation within an 11-ax framework: management and leadership, planning, training, employee management, patient management, resource management, process management as well as employee results, patient results, community results, and hospital results (25). The present model covers all the axes of the Iranian accreditation model in the form of floor and subfloor. In addition to the field in which the hospital provides services; such as hospital culture, attention to teamwork, organizational growth, and learning, location and position of the hospital in the linear system (local, provincial and national hospitals).

Although in the accreditation model, the three standards of structure, process, and outcome applied to hospital evaluation have been considered. More emphasis has been laid on the structure and process standards, and outcome standards have received less emphasis. Furthermore, the standards have put more emphasis on patient outcomes and paid little attention to the outcomes associated with employees, society, and hospital productivity.

Nippak et al. (2016) presented a Suppliers-Inputs-Process-Outputs-Customers (SIPOC) chain model to construct a visual model of activities for hospital performance evaluation (15). Nippak's model is consistent with the present model in terms of visual dimensions. In other words, the three main dimensions of input, process, and output in this model are in line with the dimensions of the present model and the only difference is the lack of attention to the control and context dimensions.

Based on the results of the present study, the patient, employee, society, and hospital performance results were considered as the main categories of the results dimension evaluation. The results of Sack et al.'s (2017) research stated that patient satisfaction measurement can be applied to obtain information regarding the structure, process, and output(26). Abedi et al.'s study (2016) showed that the most important ethical issue in a hospital is patient rights (27). The results of the research by Yarmohammadian et al. (2013) revealed that the evaluation program is mostly associated with employees and that hospital performance evaluation is affected by employees and employees are also affected by the performance evaluation (28). The results of Papanicolas et al.'s study (2008) stated that one of the main purposes of performance assessment is to inform the public about the performance of organizations, but in practice, hospitals pay less attention to it (29), which is consistent with the results of the present study. One of the differences between the current model and other common models is the balanced attention to all the consequences of hospital performance. Paying attention to patients' outcomes such as patient satisfaction and percentage of leaving with personal responsibility along with staff outcomes such as staff satisfaction, out of service and community outcomes such as hospital participation and commitment to community health promotion as well as key hospital performance indicators such as bed occupancy rate.

Based on the results of the present study, internal control and outer control were found to be effective in the hospital performance assessment. The results of Berg et al.'s (2005) and Papanicolas et al.'s (2008) illustrated the best performance system is the system that addresses all the hospital-related issues and monitors all the activities performed in this regard (29, 30) which is consistent with the results of the present study. Having a regular plan to visit different wards and units of the hospital, analyzing the results of the visit, and taking reliable measurements to improve the identified weaknesses are notable points in the present model. Also, analyzing the reports of external inspectors and using them to improve the presentation processes.

Relying on the results of the present study, hospital culture, hospital position, and the role of evaluators were found to be effective in performance assessment. Greenfield et al. (2008) recommended hospital performance is directly associated with the organizational culture (19). Therefore, the hospital evaluation model of each country must be designed based on the structure and culture of that country. Al-Assaf et al. (2017) also presented the current accreditation standards did not take into account the differences between organizations (31). Hakkak et al.(2018) identified the inability to select and train evaluators, the weakness in evaluator' knowledge and their uniform practices, lack of mastery, and different behaviors as major challenges in performance evaluation (32), which is in line with the findings of the present study. Weakness in the selection and training of evaluators, weak knowledge and uniformity of evaluators, lack of mastery and their different behavior, differences in the position of hospitals at the local, provincial and national levels to provide services to patients can be considered major challenges of current hospital evaluation systems. Therefore, the present model paid attention to these components that affect the evaluation of hospital performance. It should be noted that due to the diversity of participants in different occupational, age, and gender categories, an attempt was made to minimize diversity and generalizability limitations.

In conclusion, components like input resources, clinical, support and management processes, Outcome of patients, staff, community and hospital, in-hospital and out-hospital controls, and context components such as hospital culture, unity of evaluators, and hospital position in explanation the hospital performance appraisals are effective. It is suggested to apply the performance assessment model along with the IPOCC approach to consider hospital processes and the Output obtained from the proper implementation of these processes in all areas. The mentioned areas contain the control and context areas in which hospitals provide services, in addition to considering physical, human, financial, and equipment resources according to the traditional perspectives.

## Figures and Tables

**Figure 1:**
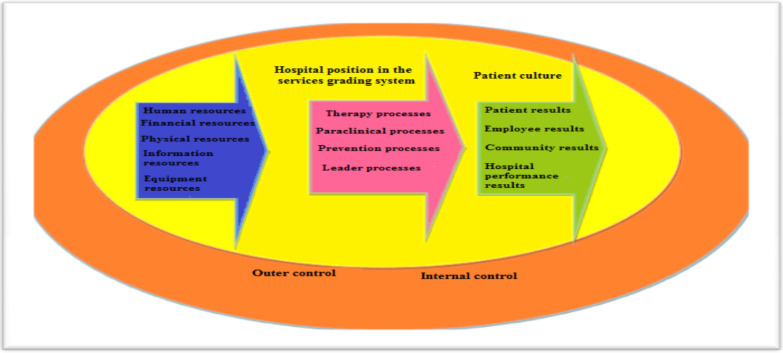
The proposed model
